# A Reflective Commentary on the Nursing of Migrants

**DOI:** 10.4274/balkanmedj.galenos.2020.2020.4.163

**Published:** 2020-10-23

**Authors:** Theofanidis Dimitrios, Fountouki Antigoni, Aysu Zekioğlu

**Affiliations:** 1 Department of Nursing, International Hellenic University, Thessaloniki, Greece; 2 Department of Health Management, Trakya University School of Health Sciences, Edirne, Turkey

## To the Editor,

Migration has been a common phenomenon for ages. Greeks are renowned for their bold and often perilous ventures to far-off lands, resulting in a diverse Greek Diaspora. This background awareness on the acceptance of its people in places where they have arrived has fueled a sympathetic consideration for others seeking refuge from wars and other calamitous conditions. This has been put to an extreme test these past few years as rescuing migrants, often from dangerous sea journeys, has stretched our hospitality to the limit.

Our borders are vulnerable to all kinds of exploitation as they comprise thousands of islands that can act as landing points and a land mass that act as a link between the Middle Eastern countries and Europe. The North African countries are not exempted as they; too, border the Mediterranean, thereby drawing migrants fleeing to Europe, often via the southern flank, which includes Greece.

With this geosocial backdrop, the recent influx of hundreds of thousands of migrants to Greece has been, and still remains, a huge challenge for local healthcare professionals and others trying to deal with such unprecedented demands. The situation has created exceptional controversies in a society with a predominantly philoxenic attitude, which has been suffering of austerity for more than a decade. This letter provides a personal reflection on the challenges that confront local citizens and the migrants who land at the Greek borders and beset by the challenges involved in the pursuit for a better life.

After a treacherous trip by sea, most migrants land on a Greek island, with an intense desire to quickly move on to the northern borders in order to reach more affluent, northern European countries. However, political and financial directives dictate that in most cases, they will stay in island encampments for a long time to come. Their immediate needs include food, shelter, clothes, and healthcare.

However, there has been heavy criticism on the part of the Greek state by various international entities for its limited intervention and offers of scarce resources made available to mismatched needs. Meanwhile, much international attention and praise has been drawn to lay Greeks who have often taken individual initiatives to alleviate the suffering of these desperate people. Examples include grandmothers who have “adopted” newborns, a baker who has been feeding large numbers of homeless, and others who have volunteered to help.

At one point, the United Nations refugee agency was concerned about the increasingly precarious situation at the border of Greece and Northern Macedonia, where force was often employed to prevent people from crossing. Many migrants failed to understand (or were intentionally misinformed) that based on a European quota for migrant dispersion within the member states, they were legally obliged to remain in their first country of entry, and that they did not have the free choice of deciding where to settle. In this context, the Office of the UN High Commissioner for Refugees (1) was particularly worried about the thousands of vulnerable refugees and migrants, especially women and children, massed on the Greek side of the border amid deteriorating conditions.

The Greek authorities were urged to improve registration and reception arrangements for people in need of international protection and to provide urgent assistance to those stranded on their side of the border. However, while helping them move to reception facilities away from it, the great majority of lay Greeks continued to offer a positive and welcoming environment (2,3).

Therefore, nurses who are at the frontlines, receiving and treating migrants, need to be alert to the possibility that up to half of the people they are dealing with may have mental disorders, often as the direct result of the three phases of migration (i.e., pre-migration trauma, migration per se, and post migration). Yet, the nurses in Europe, especially in Greece, too often lack the professional experience of dealing with those who have undergone severe systematic trauma from life events caused by a civil war or other similar conflicts.

In this context, we need to prepare ourselves professionally to empathize yet, at the same time, take the necessary steps to avoid an emotional burnout. The key word here is “resilience,” a quality that we should be able to help build in others as well asourselves.
